# Protein Profile and Simulated Digestive Behavior of Breast Milk from Overweight and Normal Weight Mothers

**DOI:** 10.3390/foods10040887

**Published:** 2021-04-18

**Authors:** Silvia Sánchez-Hernández, Laëtitia Théron, Pablo Jiménez-Barrios, Manuel Olalla-Herrera, Isidra Recio, Beatriz Miralles

**Affiliations:** 1 Departamento de Nutrición y Bromatología, University of Granada, 18071 Granada, Spain; silsanchez@ugr.es (S.S.-H.); olalla@ugr.es (M.O.-H.); 2 Institut National de Recherche Pour l’Agriculture, l’Alimentation et l’Environnement (INRAE), UR370 Qualité des Produits Animaux, F-63122 Saint Genès-Champanelle, France; laetitia.theron@inrae.fr; 3 Instituto de Investigación en Ciencias de la Alimentación, CIAL, (CSIC-UAM), Nicolás Cabrera 9, 28049 Madrid, Spain; pablo.jimenez.barrios@csic.es (P.J.-B.); i.recio@csic.es (I.R.); 4 Instituto de Investigación Biosanitaria ibs. GRANADA, University of Granada, 18071 Granada, Spain

**Keywords:** human milk, MALDI mass spectrometry, body mass index, in vitro infant digestion, bioactive proteins

## Abstract

Human milk proteins have shown to vary in concentration and distribution through lactation. However, while some regulatory components, such as hormones, have shown associations with regard to the mothers’ body mass index, there is limited information on the possible influence of this condition on the whole protein distribution. The objective of this study was to evaluate the protein profile of human milk from normal weight and overweight or obese mothers to identify differences in protein expression in colostrum, transitional and mature milk. The mass spectrometry analysis showed the ability to class with a high degree of confidence the lactation state and the milk profile according to the mother’s condition. Individual milk samples were subjected to a digestion in vitro model that takes into account the specificities of the gastrointestinal conditions of full-term newborn infants. The digestion products were compared with available data from the digestive contents in newborns. The behavior of the most abundant proteins and the overall peptide generation and survival, showed good correspondence with in vivo data.

## 1. Introduction

Human milk presents a combination of components ideal for infant nutrition, allowing optimal growth and providing several short and long-term health benefits. While lipids are the largest source of energy in breast milk, contributing 40–55% of the total energy, proteins from breast milk provide approximately 8% of the energy required by the infant [1]. However, they constitute an essential fraction from the point of view of biological implications, including antimicrobial and immunomodulatory activities, and stimulating the absorption of nutrients [2]. Hormones (such as insulin or adipokines) are contained within the peptide components and may influence energy intake, weight gain, growth and development of infants directly or indirectly [3].

Milk proteins can be classified into caseins, whey proteins and the milk fat globule membrane (MFGM) fraction. Breast milk composition, and concretely the protein fraction, varies during lactation to adapt to the newborn’s protein requirements. The total content of casein, suspended in casein micelles, and whey proteins, soluble, change profoundly over time; whey proteins dominate the profile in the first days of lactation compared to virtually undetectable casein [4]. In mature milk, an estimated average whey/casein ratio of 60:40 has been reported, although quantification with proteomics reveals a median whey/casein ratio even lower, 73:27 [5]. Regarding protein composition, the levels of α-lactalbumin (α-LA), lactoferrin and β-casein decrease throughout lactation while, lysozyme concentration increases [6]. Milk composition can also vary with mother´s phenotype and health status. Increased levels of glucose and insulin are reported in obese and overweight mothers [7]. Maternal body mass index (BMI) is positively correlated with leptin levels in milk [8]. Maternal adiposity has also been associated with increased amounts of non-glucose monosaccharides [9] and with higher milk fat and lactose, whereas total protein concentration seems to be unaffected [10,11]. However, there is limited information about a possible influence on protein or peptide distribution.

Digestion of nutrients is an essential function step for the newborn to allow normal growth and development. Both, the supply of essential amino acids and the bioactivities of milk proteins are dependent on their digestibility: some proteins act only in the intact form, others in the form of larger or small peptides formed during digestion, and some are completely digested and serve as a source of amino acids [4]. Interesting data about healthy full term infant digestion have been reported in the last years. Some works point to the role of digestion kinetics on the distinct effect of breastfed and infant formula fed infants [12]. Many factors, such as the type of food matrix (liquid/solid), pH, the enzyme secretion, peristaltic movements and emptying of the stomach need to be considered in mimicking digestion [13]. In order to set relevant infant digestive conditions, outcomes from these models should be contrasted with in vivo data, for example, with regards to protein regions resistant to digestion. Peptidomics has allowed the identification of peptides deriving from many milk proteins in the gastric and intestinal aspirates of the newborns after their mother’s milk ingestion [14,15,16].

The objective of this study was to evaluate the protein profile of a set of human milks from normal weight (NW) and overweight/obese (OW/Ob) mothers to identify differences in protein expression in colostrum, transitional and mature milk. In addition, these milk samples were subjected to an in vitro static digestion model that takes into account the specificities of the gastrointestinal conditions of full-term newborn infants. The protein digestion products have been compared with available data from the digestive contents in newborns. The protein fraction of milk at diverse stages of lactation and the peptides released after digestion have been used to evaluate the influence of the mother’s condition on its composition.

## 2. Materials and Methods

### 2.1. Subjects and Samples

Human milk samples were provided by participating mothers in a study conducted in the Obstetrics and Gynecology Department of “Hospital Universitario Virgen de las Nieves” from Granada (Spain). Ethics were approved by the relevant scientific committee and the trial was registered at ClinicalTrials.gov (accessed on 15 April 2019) (number NCT): NCT02811172. Details of the study were explained to all mothers who voluntarily gave written consent to participate. Eighteen lactating women were recruited for HM collection, with each mother donating three samples (colostrum, transitional and mature milk). The main characteristics of sampled mothers are shown in Table 1. Colostrum corresponded to milk from the 3rd to the 5th post-partum day, transitional milk to days 9–13 and mature milk from day 17 thereafter. Mothers with BMI in the 18.5–24.9 kg m^−2^ range were included in the NW group while those with a BMI greater than 25.0 kg m^−2^ were included in the OW/Ob group. All human milk samples collected from the participants in sterile tubes were aliquoted and immediately stored at −80 °C until used.

### 2.2. MALDI-TOF Protein Profile

Milk was centrifuged at 11,000× *g* for 20 min at 4 °C for skimming. The skimmed milk was subsequently diluted 1:100 with ultrapure water (Milli-Q Millipore) and subjected to mass spectrometry analysis. An aliquot (1 µL) of skimmed milk was directly spotted onto an MSP 96 polished steel target (Bruker Daltonics, Bremen, Germany), overlaid with 1 µL of sinapinic acid matrix, in 50% acetonitrile, containing 0.1% trifluoroacetic acid (*v*/*v*) and allowed to dry at room temperature, and analyzed on an Autoflex speed instrument (Bruker Daltonics, Bremen, Germany) using previously reported conditions [17]. Three independent spectra for each skimmed milk’s fraction were collected in the automated mode, externally calibrated by using the Bacterial Test Standard (Bruker Daltonics, Bremen, Germany) and subsequently analyzed with the FlexAnalysis version 3.3 (Bruker Daltonics, Bremen, Germany) for the quality checking on raw data.

MALDI-TOF spectra were analyzed with ClinProTools (Version 2.2, Bruker Daltoics, Bremen, Germany) for statistical treatment. Baseline was subtracted using the Top Hat algorithm with 10% of minimal baseline width, and spectra were smoothed using the Savitsky–Golay algorithm with one cycle of 5 *m*/*z* width. Peak picking was calculated on total average spectrum with a signal-to-noise of 3 and 5% of relative threshold base peak. Principal component analysis (PCA) was calculated to visualize samples projection on the score plot and the variables explaining the projection of the loading plot. Supervised analysis consisted in model calculation with three complementary algorithms. The quick classifier (QC) algorithm uses the statistical differences between classes to discriminate them. The peaks included in the calculation were thus selected according to their *p*-value after an automatic detection of peak number. The supervised neural network (SNN) algorithm is an iterative method based on the characteristics of data distribution [18]. The number of peaks involved in the calculation and the number of prototype detection were determined automatically by the algorithm. The genetic algorithm (GA) is a random algorithm that mimics the natural evolution [19]. Each spectrum is considered as a chromosome and each peak as a gene, and the overall principle is to recover the family belonging, meaning the class discrimination. For these three models, internal validation was calculated to ensure the calculation reliability, using 20% of the spectral data selected randomly and 10 iterations.

### 2.3. In Vitro Simulated Gastrointestinal Digestion

The infant gastrointestinal static in vitro model was carried out following the protocol described by Ménard et al. [20]. The gastrointestinal in vitro simulation included two consecutive steps: a gastric phase (60 min at 37 °C with pepsin and lipase at pH 5.3) and an intestinal phase (60 min at 37 °C with porcine pancreatin and bile at pH 6.6). Enzyme activity and bile concentration were measured according to the assays described by Brodkorb et al. (2019) [21]. Pepsin and gastric lipase were added as rabbit gastric extract (RGE). The added amount of RGE covered 100% of lipase activity and 120% of pepsin activity (320 U/mL). Samples were withdrawn at 5, 15, 30 and 60 min during gastric digestion and the reaction was stopped by adjusting the pH at 7.0 with NaOH 1 M and snap freezing in liquid nitrogen. The intestinal phase was carried out by mixing the final gastric volume with an appropriate amount of simulated intestinal fluid, containing pancreatin from porcine pancreas, which covered the lipase (90 U/mL) and trypsin (16 U/mL) activity required of intestinal content, and porcine bile extract 3.1 mM and the pH was adjusted to the intestinal pH of 6.6 using HCl 1 M. Samples were withdrawn at 5, 20 and 60 min during intestinal digestion and the reaction was stopped by snap freezing in liquid nitrogen. All samples were lyophilized and stored at −20 °C until analysis. Nitrogen contents at the end of the gastric and intestinal phase were determined by elemental analysis.

### 2.4. Sodium Dodecyl Sulphate-Polyacrylamide Gel Electrophoresis (SDS-PAGE) and Identification of Bands by In-Gel Digestion

Undigested (control) and digested human milk samples were dissolved at 1 mg of protein/mL in sample buffer and analyzed on 12% Bis-Trispolyacrilamide gels (Criterion_XT, Bio-Rad, Hercules, CA, USA). In gel digestion and analysis on an Autoflex speed MALDI-TOF/TOF (Bruker Daltonics, Bremen, Germany) instrument was performed as previously reported [22]. MASCOT v2.4 software (MatrixScience, Boston, MA, USA) was used to carry out protein identification searches against a homemade database of human milk proteins selected from the Uniprot database (https://www.uniprot.org/ accessed on 15 April 2019).

### 2.5. Analysis by Ultra-Performance Liquid Chromatography (UPLC)

Human milk proteins were separated by reverse-phase UPLC following an adaptation from Visser et al. (1991) [23] on an Aquity UPLC (Waters Technologies, Cerdanyola del Vallès, Spain). Solvent A was acetonitrile-water-trifluoroacetic acid (100:900:1 *v*/*v*/*v*) and solvent B was the same mixture with the proportions 900:100:0.1 (*v*/*v*/*v*). The analytical column was a Phenomenex AerisTM 3.6 μm Widepore XB-C18 (150 mm × 2.1 mm). Briefly, 100 µL of human milk or digested sample were mixed with 100 µL of 0.02 M 1,3-bis[tris(hydroxymethyl)methylamino] propane (Bis-Tris) buffer (pH 7) containing 8 M urea and 0.3% of 2-mercaptoethanol. After standing at room temperature for 1 h, samples were diluted (four-fold for human milk/gastric samples and two-fold for intestinal samples) with solvent A containing 6 M urea, and centrifuged at 13,000× *g* 5 min before injection of the supernatant.

### 2.6. Analysis of Digests by HPLC–Tandem Mass Spectrometry (HPLC–MS/MS)

Freeze-dried samples were reconstituted in ammonium carbonate (NH_4_CO_3_) 25 mM and treated for 60 min at 37 °C with 1,4-dithiothreitol 140 mM (1:1, *v*/*v*) (Sigma-Aldrich, St. Louis, MO, USA) to reduce disulfide linkages in order to improve the identification. Samples were analyzed by HPLC–MS/MS in duplicate using an Agilent 1100 HPLC system (Agilent Technologies, Waldbron, Germany), equipped with a Mediterranean Sea18 column (150 mm × 2.1 mm, Teknokroma, Barcelona, Spain) connected to an Esquire 3000 linear ion trap mass spectrometer (Bruker Daltonics GmbH, Bremen, Germany) as previously described [24]. The spectra were recorded over the *m*/*z* ranges 100–700, 100–1700 and 100–2000, selecting 500, 750 and 1200 as target mass, respectively. A homemade database of most abundant human milk proteins was used for the peptide sequencing in MASCOT v2.4 software. No specific enzyme cleavage was used. Peptide mass tolerance was set to 0.1% and 0.5 Da for MS and MS/MS analysis, respectively. Biotools version 3.2 (Bruker Daltonics, Bremen, Germany) was used for the interpretation of the matched MS/MS spectra. The list of peptides appearing in the selected number of subjects was compiled through Venn diagrams using the Venny tool [25]. The representation of the peptide profile was performed with Peptigram [26]. Bioactive sequences were searched in the MPDB (Nielsen, New York, NY, USA). With the identified peptides after simulated digestion, hierarchical clustering trees were built and optimized using the PermutMatrix software version 1.9.3.0 [27]. The mean center rows were used as datasets, and Ward’s dissimilarity aggregation procedure based on Pearson distance was used.

## 3. Results and Discussion

### 3.1. Protein Profile of Human Milks by MALDI-TOF Mass Spectrometry

**Figure 1:**
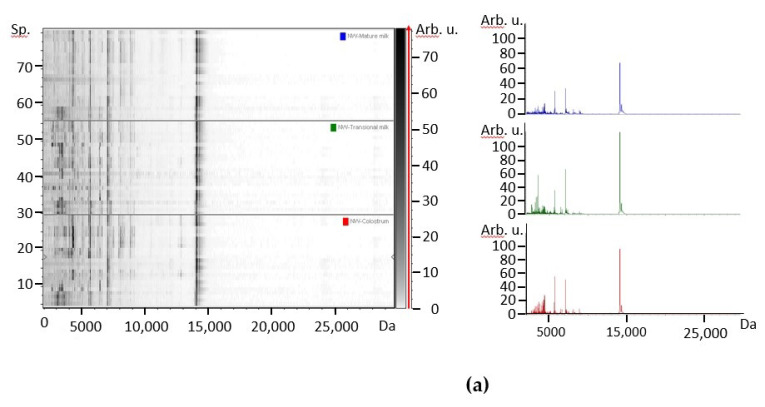

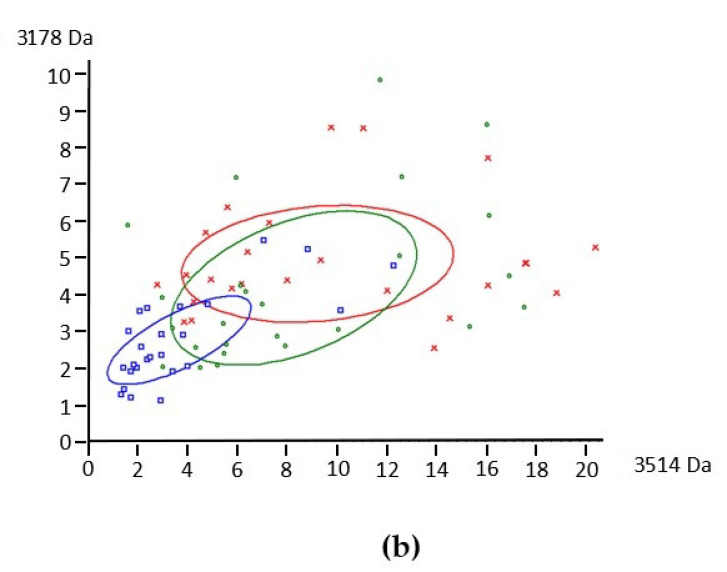
(**a**) Gel-like view and average spectrum. (**b**) 2D-plot protein spectra according to the intensities of *m*/*z* 3178 and *m*/*z* 3514 from colostrum (red), transitional milk (green) and mature milk (blue) from NW mothers. Number of points = 72 (8 subjects, 3 milk types, 3 replicates).

**Figure 2:**
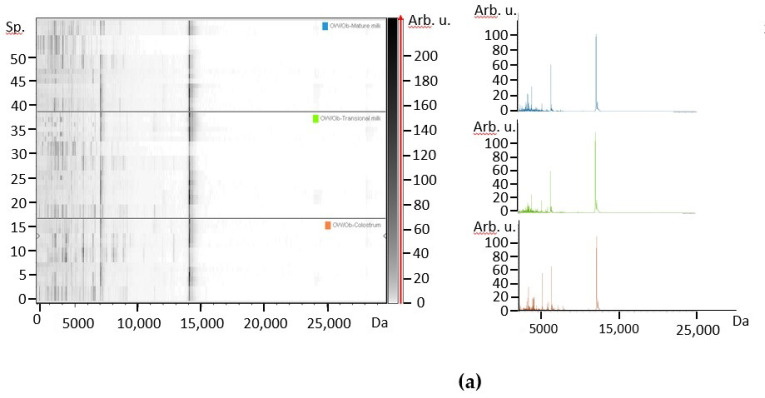

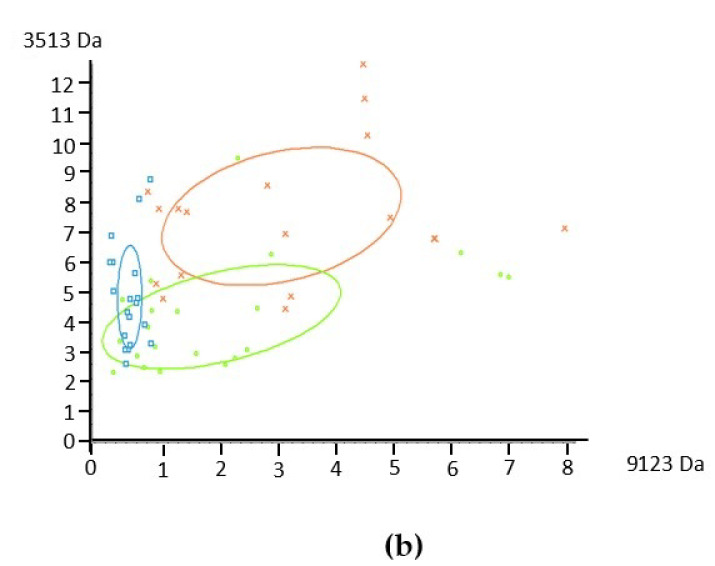
(**a**) Gel-like view and average spectrum. (**b**) 2D-plot protein spectra according to the intensities of *m*/*z* 3513 and *m*/*z* 9123 from colostrum (light red), transitional milk (light green) and mature milk (light blue) from OW/Ob mothers. Number of points = 54 (6 subjects, 3 milk types, 3 replicates).

The profile of individual milks counting large peptides and small proteins was examined by MALDI-TOF mass spectrometry. The spectra from colostrum, transitional milk and mature milk from mothers corresponding to the different BMI groups, NW (Figure 1) and OW/Ob subjects (Figure 2) were first contrasted. In the gel-like view of human colostrum, transitional milk and mature milk of all individual spectral data are shown as lines and the grey level represents the peak intensity, which permits to visualize individual variability. In contrast, the spectrum view represents the average profile from all samples. In the protein profile, the main peak around 14 kDa corresponds to α-LA, although adjacent small signals can be ascribed to lysozyme C and other less abundant proteins in human milk. Besides, many peaks in the range 2000–10,000 Da characterize all samples, although species between 3000 and 4500 Da were more intense in colostrum and transitional milk. In mature milks, peak intensity was comparatively lower, which is compatible with inferior relative protein content. It is known that the protein content of human milk decreases rapidly during the first month of lactation, and declines more slowly after that [4,28]. In spite of the use of sinapinic acid matrix, which favors the ionization of high molecular weight proteins, the intensity of peaks above *m*/*z* ratio of 15,000, where caseins should appear, was very low. An impaired detection of higher mass-range species in human milk under 30 days post-partum was previously reported under similar conditions [17].

The effect of the lactation time in the protein profile was assessed by studying samples of colostrum, transitional milk and mature milk from 8 NW and 6 OW/Ob mothers. A representation of the three milk types is shown for each group in the form of 2D-plot according to the two peaks with highest variance (Figure 1b or Figure 2b). Consistent with other measurements, the interindividual variation is considerable, although dispersion was lower for mature milk compared to both colostrum and transitional milk. However, an evolution from colostrum to mature milk can be observed in both groups. Less spreading was observed in the OW/Ob group, in spite of a lower number of subjects.

**Figure 3:**
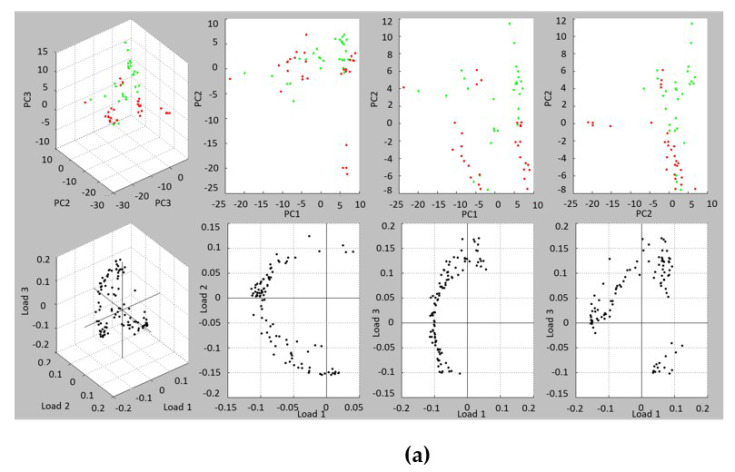

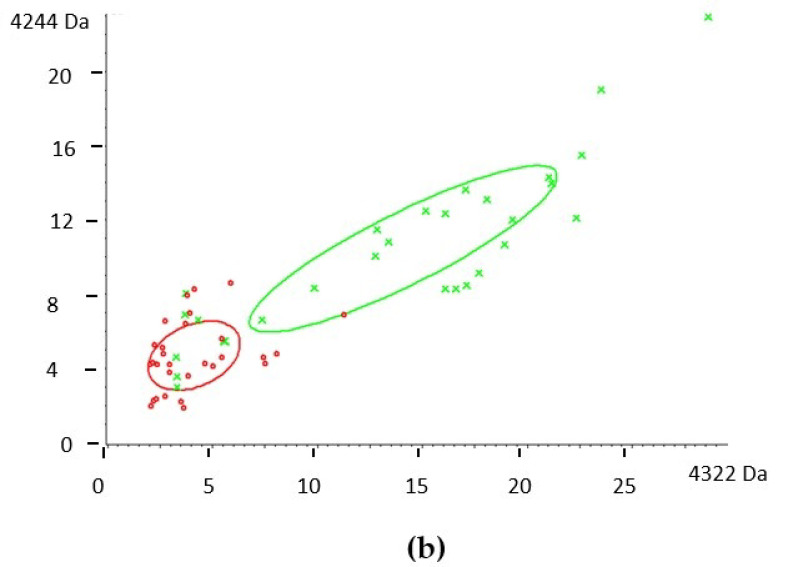
(**a**) PCA analysis contrasting spectral data of mature milks from NW (green) and OW/Ob (red) subjects. 3D view and combinations of the first three principal components. (**b**) 2D-plot protein spectra according to the intensities of *m*/*z* 4244 and 4322. Number of points = 54 (9 NW subjects, 9 OW/Ob subjects, 3 replicates).

A second design was used to study the effect of the mother BMI condition in the protein profile of the expressed mature milk. Combined spectra from 9 NW and 9 OW/Ob subjects were used to determine the state of classification in the first three principal components (PC) (Figure 3). The PCA analysis showed that the mature milk profiles clustered together, with certain individuals from both classes being slightly separated. The contributions of PC1, PC2 and PC3 to the generation of profile explained less than 30% of the variance. However, the 2D-plot representation according to the intensities of *m*/*z* 4244 and *m*/*z* 4322 grouped OW/Ob and NW separately, with a tighter grouping in the case of OW/Ob mothers. These two masses corresponded to well defined signals present in all samples and merit attention as possible biomarkers. A predictive model generation with different algorithms was used to evaluate the discrimination potential of the protein profile. The QC algorithm provided 83.44% recognition capability with a cross validation of 76.61%, the signal corresponding to mass 4322 being selected in the integration region for classification. The application of the models based on the SNN and GA provided higher rates, with cross validation values of 79.77 and 78.98%, and recognition capability percentages of 96.49 and 98.21%, respectively, the GA providing 100% recognition capability for class 2 (OW/Ob). The list of masses involved in the model generation and their weight are shown in Table S1.

This supervised analysis showed the ability of MALDI-TOF analysis to class with a high degree of confidence mature milks according to the mothers BMI. Recent studies relating breast milk with body composition have indicated that individual bioactive components of human milk may regulate different compartments of infant weight gain, i.e., adiposity or accumulation of lean mass, separately [29]. In fact, some studies correlate the adipokines content in breast milk with the respective levels in maternal and infant blood [30]. In this regard, it is still unknown how alterations in breast milk composition will subsequently impact later health outcomes. Delivered components as different as proteins, fatty acids or insulin have shown associations with infant fat mass gain [31]. The used strategy has allowed to evidence specific signals characterizing the mothers by BMI. Therefore, they might be useful to identify new biomarkers and complement these analyses in the way to discern the role of human milk composition in the infants’ development.

### 3.2. Protein Degradation of Human Milk during Infant In Vitro Gastrointestinal Digestion

**Figure 4:**
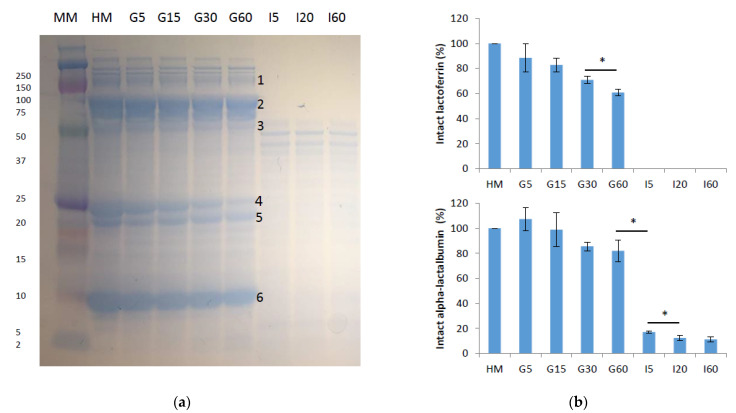
(**a**) SDS-PAGE profile of a mature milk sample submitted to in vitro gastrointestinal digestion. Band identification by tandem MS: 1. Bile salt-activated lipase. 2. Lactoferrin. 3. Immunoglobulin heavy constant alpha 4. β-casein. 5. β- and αs1-casein. 6. α-LA. (**b**) Quantification of lactoferrin and α-LA by UPLC (*n* = 4). * *p* < 0.05 (*t*-test) MM: molecular marker; HM: human milk; gastric digestion time 5 (G5), 15 (G15), 30 (G30) and 60 (G60) min; intestinal digestion time 5 (I5), 20 (I20) and 60 (I60) min.

Ten mature milk samples, six from NW subjects and four from OW/Ob subjects, were submitted to in vitro gastrointestinal digestion under conditions that simulate those of the newborn. The SDS-PAGE electrophoretic pattern during gastrointestinal digestion of a milk sample from a NW donor is presented in Figure 4a. Digested samples from the gastric (G5, G15, G30 and G60) and intestinal (I5, I30 and I60) phases were compared with the undigested human milk (HM). The analysis of the gel bands by MALDI–MS/MS allowed identification of bile salt-activated lipase (BAL), lactoferrin, immunoglobulin heavy constant alpha 2 (IgHA2), β-casein and α-LA. The whey proteins α-LA and lactoferrin are major proteins in human milk, comprising 25–35% and 15–20% of the total protein content, respectively [32], as observed in the band size. Despite similar molecular weight than lactoferrin, 78 vs. 79 kDa, BAL shows a lower electrophoretic mobility, probably due to its mucin-like structure with extended conformation [33]. α-LA and lactoferrin resisted gastric digestion at G60 due to the globular and compact structure. The caseins, especially β-casein, were hydrolyzed in some measure over gastric digestion, in accordance with their loose and flexible structure that makes them susceptible to pepsinolysis. All proteins were detected after 60 min, consistent with the high gastric pH in infants (5.3) compared to the adult (3.0) that causes pepsin only be at 10% of its maximal activity. By contrast, intestinal digestion resulted in a rapid hydrolysis of the intact proteins remaining after the gastric step. After five min, the bands visible on the SDS-PAGE gel corresponded to the proteins added in the pancreatin, as previously observed [20].

Human milk samples and in vitro digests were analyzed by UPLC to quantitatively determine the protein changes over digestion. Lactoferrin remained as 61% intact protein during the gastric phase with no protein being detected in the intestinal phase. 82% intact α-LA was observed, while around 11% of the initial concentration was determined at the end of the intestinal digestion (Figure 4b). These results are similar to the percentage of α-LA reported to be resistant to hydrolysis in the development of this digestion model (86.7%), although an infant formula with bovine proteins was used [20]. Interestingly, De Oliveira et al. (2017) [34] determined the proteolysis of human milk proteins during gastric digestion of human milk by preterm infants. In that study, ca. 40% and 75% of lactoferrin and α-LA, respectively, was found in the infant stomach after 90 min of ingestion, in good correspondence with the present values. The observed protein resistance might well mimic the full-term infant digestion, which is intended by this protocol, although full-term infant data of intact protein resistance should be contrasted when available.

Lactoferrin and IgA have a structure that makes them comparatively resistant to digestion. Furthermore, intact lactoferrin and IgA have been observed in stools of both preterm and term exclusively breastfed infants by immunological methods [35]. It is considered that only partial digestion of human lactoferrin takes place in the infant and this supports the beneficial effect of this abundant iron-binding protein in terms of bacteriostatic, bactericidal and antiviral activity. This report shows resistance of human lactoferrin under these gastric digestive conditions, in good correspondence with previous in vivo data. On the other hand, when comparing digestion of different human Ig in a dynamic simulated model, higher resistance of IgA and IgM has been reported compared to IgG, by means of proteomic techniques [36].

### 3.3. Peptidomic Characterization of In Vitro Digests

**Figure 5:**
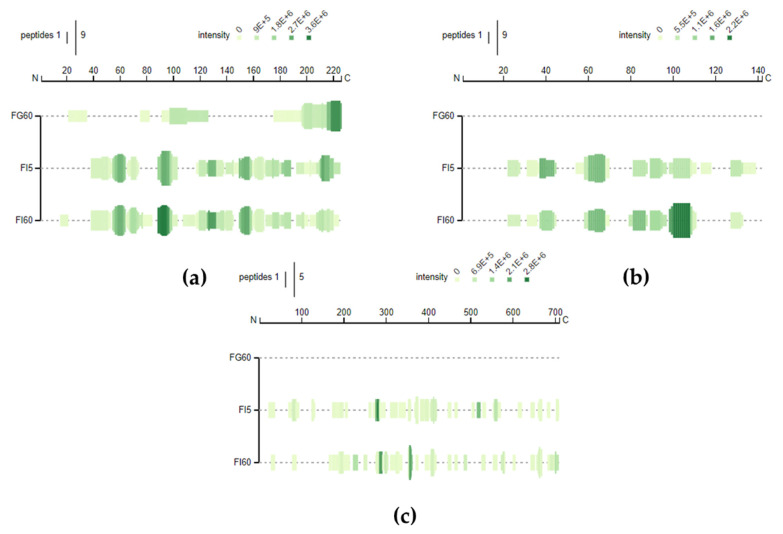
Peptigram profile of β-casein (**a**), α-LA (**b**) and lactoferrin (**c**) derived peptides in digests of human milk (*N* = 10) after 60 min gastric digestion (FG60), and 5 min (FI5) and 60 min (FI60) intestinal digestion.

Human milk gastrointestinal digests were analyzed under equal conditions by HPLC–MS/MS. The peptide trace from α-LA, β-casein and lactoferrin was represented using the Peptigram tool. Figure 5 represents the peptides identified after the application of the gastric phase and the intestinal phase at both 5 and 60 min. After the gastric digestion, only peptides from β-casein were identified, consistent with the resistance of α-LA and lactoferrin. Gastrointestinal digests produced a pattern with higher number of peptides, with great similarity between 5 and 60 min. However, more intense color in specific areas denotes greater number of overlapping identified peptides after 60 min for the three proteins. Calculated coverages reached 86, 52 and 48% for β-casein, α-LA and lactoferrin, respectively.

The peptides identified in the human milk gastrointestinal digests (endpoint at 60 min) consistently derived from 20 proteins (Table 2). These proteins were considered representative because a similar number of released peptides were found in at least four subjects. Identified peptides arose, for the most part, from β-casein, α-LA and lactoferrin, in accordance with their abundance in milk. Other predominantly precursor proteins were butyrophilin subfamily 1 member A1, BAL and IgAH2. From the human milk fat globule membrane, mucin-1, lactadherin and butyrophilin are three major components that gave rise to peptides under the used digestive conditions. It has been reported that mucin and lactadherin resist digestion in the stomach of milk-fed infants, while butyrophilin is rapidly degraded [37]. The number of peptides from the last protein in the digests can be ascribed to this rapid degradation.

The identified sequences were contrasted to those previously found upon analysis of the gastrointestinal tract content in infants [14,15,38] or in a suckling rat pup model [39], after human milk consumption. Ninety sequences from seven proteins found upon in vitro digestion were coincident with those reported in vivo. The complete list of identical peptides to those found in vivo is shown in Table S2.

**Figure 6:**
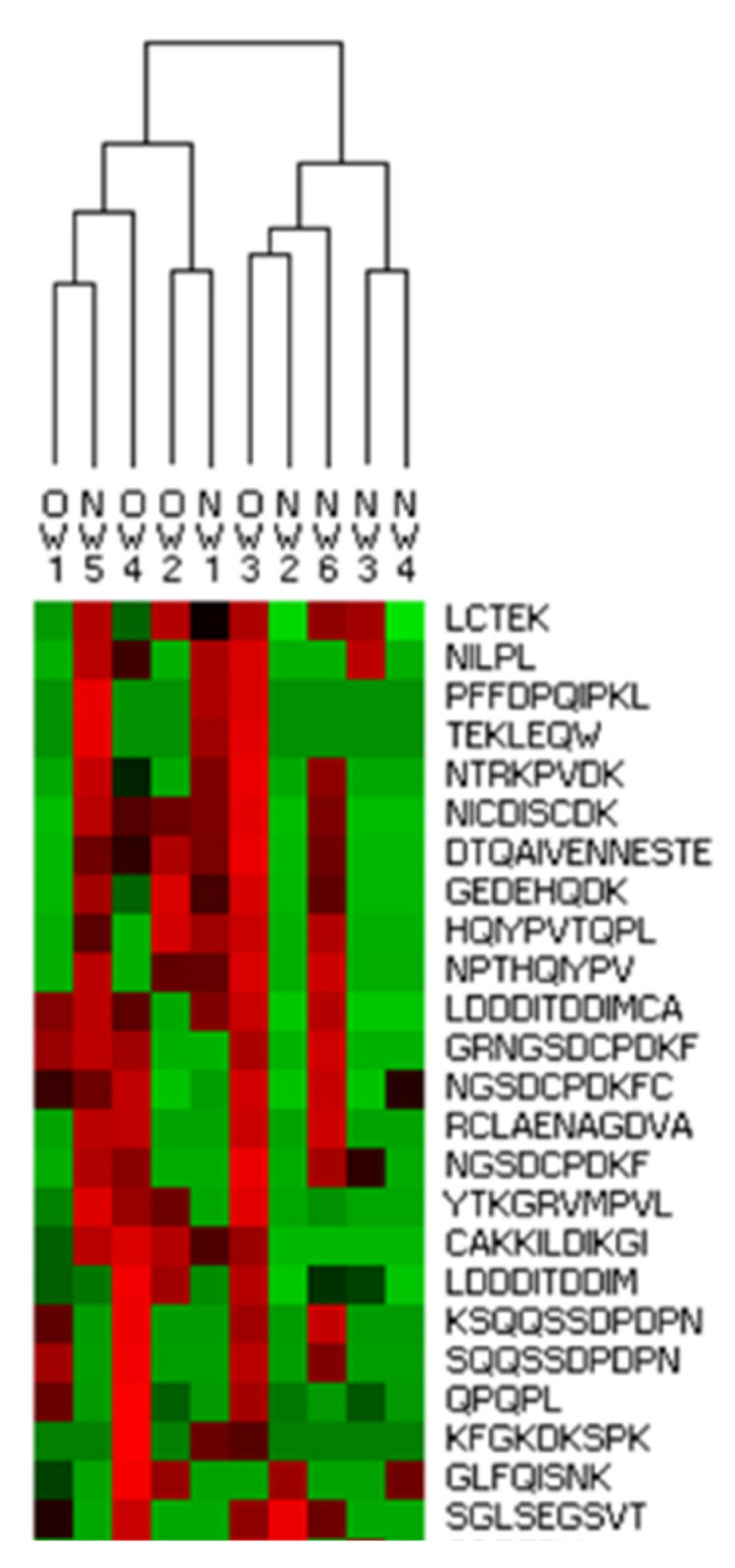
Heat map representation (fragment) of the hierarchical clustering analysis of peptides identified after in vitro digestion of breast milk. Columns correspond to subjects included in the overweight/obese group or normal weight group. Rows correspond to intensity of sequences derived from β-casein, α-lactalbumin and lactoferrin identified in at least 3 subjects. Complete figure in Figure S1.

The results from the individual digestions in terms of sequence and intensity of identified peptides were analyzed with software permitting the visualization of the possible associations. Identified peptides derived from α-LA, lactoferrin and β-casein were used to construct a dendrogram (Figure 6). The peptide fingerprint from digests did not discriminate the samples by the donor BMI, indicating that the peptide profile after in vitro digestion reduces the differences observed in the protein fraction. On one side, this can be attributed to digestion bringing closer the protein features of differing milk classes. On the other side, it might also be the case that resistant peptides are not those responsible for the differences. Discriminating peptides might have been degraded, or be too large to be detected by the proteomic approach employed. Lastly, identical (in vitro) gastrointestinal conditions have been applied to the samples but the impact of differing milk composition on the individual digestive behavior is still unknown and is worthy of future studies.

Although the number of subjects is acknowledged as a limitation, this study provides novel tools to depict the protein/peptide fraction of human milk, which can be used in nutritional studies intended to associate milk composition with the physiological status while nursing.

## 4. Conclusions

Human milk contains a multitude of proteins, with a dynamic composition varying along lactation time. The applied MALDI-TOF analysis has proved effective as a tool to associate the effect of the lactation stage with the protein profile of the expressed milk in both NW and OW/Ob subjects, lower dispersion being found in the second group. Moreover, the mothers BMI could be predicted using the variations in the 4322 *m*/*z* signal. The study of a larger amount of subjects might contribute to identify biomarkers making it possible to associate the breast milk composition with metabolic features in the mother and later health outcomes in the offspring. The newborn digestion model results in a resistance of most abundant human whey proteins, α-LA and lactoferrin, similar to the observed in vivo. Likewise, proteins ascribable to the undegraded peptides and a large number of specific sequences closely resemble those previously found in newborn digests after human milk consumption. Therefore, important outcomes after passage of human milk through the gastrointestinal tract can be predicted using the infant model. It remains to elucidate if the different protein composition might have physiological significance for the newborn.

## Figures and Tables

**Table 1:** Demographic description of study mothers and delivery characteristics by group.

Characteristics	Normal Weight ^a^ (*N* = 9)	Overweight/Obese ^a^ (*N* = 9)	*p*-Value ^b^
Age, years	33.0 (5.5)	32.0 (5.0)	0.8642
Colostrum collection, days postpartum	4.0 (1.0)	5.0 (1.0)	0.1340
Transitional milk collection, days postpartum	9.5 (1.0)	10.3 (1.3)	0.1970
Mature milk collection, days postpartum	19.0 (3.0)	19.0 (0.8)	0.6445
Gestational age, days	278.0 (14.0)	282.0 (10.8)	0.2162
BMI early pregnancy, kg m^−2^	21.45 (1.36)	30.53 (7.88)	0.0012
BMI at delivery, kg m^−2^	25.95 (2.39)	34.23 (3.64)	0.0047
Weight gain, kg	12.0 (4.5)	11.5 (4.5)	0.7576
Newborn weight, kg	3.23 (0.40)	3.44 (0.38)	0.2343
Gender distribution, %			
Female	43	50	
Male	57	50	

BMI: Body Mass Index; ^a^ Median (Interquartile range) ^b^ Mann–Whitney test.

**Table 2:** Number identified of peptides and corresponding proteins after individual in vitro digestion of breast milk from NW (*N* = 6) and OW/Ob (*N* = 4) subjects.

Protein Name	Accession Number	Peptides Identified in Two or More Subjects (*N*)	Mol. Weight, Parent Protein (kDa)	Sequence Length, Parent Protein	Score (Digest Matches)
β-casein	P05814	123	25.38	226	332.54
α_s1_-casein	P47710	26	21.67	185	46.73
κ-casein	P07498	11	20.31	182	36.41
Lactotransferrin	P02788	121	78.18	710	103.31
α-lactalbumin	P00709	61	16.23	142	277.99
Bile Salt-activated lipase	P19835	19	79.32	753	30.53
Immunoglobulin heavy constant alpha 2	P01877	18	36.59	340	27.96
Butyrophilin subfamily 1 member A1	Q13410	23	58.96	526	28.9
Polymericimmunoglobulin receptor	P01833	25	83.28	764	37.73
Tenascin	P24821	59	240.85	2201	29.5
Mucin-4	Q99102	56	231.52	2169	38.42
Xanthine dehydrogenase/oxidase	P47989	44	146.42	1333	23.27
Receptor tyrosine-proteinkinase erbB-4	Q15303	29	146.81	1308	28.3
Osteopontin	P10451	19	35.42	314	24.96
Cadherin-1	P12830	12	97.46	882	28.02
Clusterin	P10909	11	52.50	449	25.23
Galectin-3-binding protein	Q08380	9	65.33	585	24.74
Mucin-1	P15941	8	122.10	1255	28.77
Zinc-alpha-2-glycoprotein	P25311	4	34.26	298	20.67
Plateletglycoprotein 4	P16671	3	53.05	472	22.34

## Supplementary Materials

The following are available online at https://www.mdpi.com/article/10.3390/foods10040887/s1, Table S1: MALDI-TOF signals provided by the predictive model generation. Effect of the mother condition (NW vs OW/Ob), Table S2: Identified peptides matching sequences previously observed in the gastrointestinal content of newborns or a rat pup model after human milk consumption, Figure S1: Complete heat map representation of the hierarchical clustering analysis of peptides identified after in vitro digestion of breast milk. Columns correspond to subjects included in the overweight/obese group or normal weight group. Rows correspond to intensity of sequences derived from β-casein, α-lactalbumin and lactoferrin identified in at least 3 subjects.
