# Supplementary material for: Protein Profile and Simulated Digestive Behavior of Breast Milk from Overweight and Normal Weight Mothers
Source: Foods. 2021 Apr 18;10(4):887. doi: 10.3390/foods10040887 (PMC8074095; doi:10.3390/foods10040887)
Supplement: Supplementary file 1 [file foods-10-00887-s001.zip › foods-1179562-supplementary.pdf]

Table S1. MALDI-TOF signals provided by the predictive model generation. Effect of the mother condition (NW vs OW/Ob).

Algorithm	m/z	weight
Quick classifier	4322.43	1.895
Supervised neural network	4041.4	0.123
	14281.32	0.103
	4162.71	0.097
	10516.64	0.089
	8131.44	0.066
	8838.71	0.058
	9127.24	0.052
	15203.42	0.039
	5867.56	0.038
	6294.35	0.032
	7144.01	0.028
	8558.49	0.025
	11301.63	0.025
	8420.52	0.024
	8459.07	0.024
	8245.42	0.021
	14488.11	0.021
	26114.9	0.017
Genetic algorithm	4162.71	0.687
	8838.71	1.035
	10308.53	0.432
	11960.16	0.440
	6021.39	0.129
	4162.71	0.687

Table S2. Identified peptides matching sequences previously observed in the gastrointestinal content of newborns or a rat pup model after human milk consumption.

Sequence	Range[1]	Protein[2]	*In vivo* occurrence
RETIESL	1 - 7	CASB_HUMAN	Nielsen et al (2018)
VKHEDQQQGEDEHQDK	24 - 39	CASB_HUMAN	Nielsen et al (2018)
VKHEDQQQGEDEHQDKIYPS	24 - 43	CASB_HUMAN	Nielsen et al (2018)
HEDQQQGEDEHQDK	26 - 39	CASB_HUMAN	Nielsen et al (2018)
GEDEHQDKIYP	32 - 42	CASB_HUMAN	Nielsen et al (2018)
VEPIPY	54 - 59	CASB_HUMAN	Wada et al (2014)
GFLPQNILP	60 - 68	CASB_HUMAN	Su et al (2017), Nielsen et al (2018)
AQPAVVLPVPQPE	70 - 82	CASB_HUMAN	Wada et al (2017)
QPAVVLPVPQPE	71 - 82	CASB_HUMAN	Wada et al (2017)
QPAVVLPVPQPEIMEVPK	71 - 88	CASB_HUMAN	Dallas et al (2014), Nielsen et al (2018).
VVLPVPQP	74 - 81	CASB_HUMAN	Nielsen et al (2018)
VVLPVPQPE	74 - 82	CASB_HUMAN	Nielsen et al (2018), Wada et al (2017)
VVLPVPQPEIM	74 - 84	CASB_HUMAN	Dallas et al (2014), Nielsen et al (2018), Wada et al (2017)
VVLPVPQPEIMEVPK	74 - 88	CASB_HUMAN	Nielsen et al (2018), Wada et al (2017)
VVLPVPQPEI	74 - 83	CASB_HUMAN	Dallas et al (2014), Nielsen et al (2018), Wada et al (2017)
LPVPQPE	76 - 82	CASB_HUMAN	Wada et al (2017)
LPVPQPEIM	76 - 84	CASB_HUMAN	Dallas et al (2014), Nielsen et al (2018)
LPVPQPEIMEVPK	76 - 88	CASB_HUMAN	Dallas et al (2014), Nielsen et al (2018), Dingess et al (2017), Wada et al (2017)
PVPQPEIM	77 - 84	CASB_HUMAN	Dallas et al (2014), Nielsen et al (2018)
PQPEIMEVPK	79 - 88	CASB_HUMAN	Nielsen et al (2018)
PEIMEVPK	81 - 88	CASB_HUMAN	Nielsen et al (2018)
MEVPK	84 - 88	CASB_HUMAN	Wada et al (2017)
MPVLKSPTIPFFD	100 - 112	CASB_HUMAN	Nielsen et al (2018)
VLKSPTIPFFD	102 - 112	CASB_HUMAN	Nielsen et al (2018)

LKSPTIPF	103 - 110	CASB_HUMAN	Nielsen et al (2018)
SPTIPFFDPQIPK	105 - 117	CASB_HUMAN	Dallas et al (2014), Su et al (2017), Nielsen et al (2018), Wada et al (2017)
PFFDPQIPK	109 - 117	CASB_HUMAN	Nielsen et al (2018)
PFFDPQIPKL	109 - 118	CASB_HUMAN	Nielsen et al (2018)
FDPQIPK	111 - 117	CASB_HUMAN	Nielsen et al (2018), Wada et al (2017)
DPQIPK	112 - 117	CASB_HUMAN	Wada et al (2017)
LTDLENLHLPLP	118 - 129	CASB_HUMAN	Nielsen et al (2018), Wada et al (2017)
LENLHLPLP	121 - 129	CASB_HUMAN	Dallas et al (2014), Nielsen et al (2018), Wada et al (2017)
ENLHLPLPL	122 - 130	CASB_HUMAN	Su et al (2017), Nielsen et al (2018)
HLPLP	125 - 129	CASB_HUMAN	Wada et al (2014)
MQQVPQPIPQT	135 - 145	CASB_HUMAN	Dallas et al (2014), Nielsen et al (2018)
QQVPQPIPQ	136 - 144	CASB_HUMAN	Dallas et al (2014), Nielsen et al (2018)
QQVPQPIPQT	136 - 145	CASB_HUMAN	Dallas et al (2014), Su et al (2017), Nielsen et al (2018)
VPQPIPQT	138 - 145	CASB_HUMAN	Dallas et al (2014)
LPPQPL	148 - 153	CASB_HUMAN	Wada et al (2017)
LPPQPLWSVP	148 - 157	CASB_HUMAN	Wada et al (2017)
WSVPQPK	154 - 160	CASB_HUMAN	Su et al (2017), Nielsen et al (2018), Wada et al (2014)
VPYPQ	169 - 173	CASB_HUMAN	Wada et al (2014)
LLNQELL	182 - 188	CASB_HUMAN	Nielsen et al (2018)
NQELLLNPT	184 - 192	CASB_HUMAN	Nielsen et al (2018), Wada et al (2017)
LLLNPTHQIY	187 - 196	CASB_HUMAN	Dallas et al (2014), Nielsen et al (2018)
LNPTHQIYPV	189 - 198	CASB_HUMAN	Nielsen et al (2018), Wada et al (2017)
HQIYPVTQPL	193 - 202	CASB_HUMAN	Wada et al (2017)
HQIYPVTQPLAPV	193 - 205	CASB_HUMAN	Wada et al (2017)
QIYPVTQPLAPV	194 - 205	CASB_HUMAN	Wada et al (2017)
YPVTQPLAPV	196 - 205	CASB_HUMAN	Wada et al (2017)
YPVTQPLAPVHNPIS	196 - 210	CASB_HUMAN	Nielsen et al (2018)
PVTQPLAPVH	197 - 206	CASB_HUMAN	Dallas et al (2014), Nielsen et al (2018)

APVHNPI	203 - 209	CASB_HUMAN	Wada et al (2017)
CAVSQPEAT	9 - 17	TRFL_HUMAN	Wada et al (2017)
GPPVSC	31 - 36	TRFL_HUMAN	Wada et al (2017)
FPNLC	166 - 170	TRFL_HUMAN	Wada et al (2017)
EDLSDEAERDE	216 - 226	TRFL_HUMAN	Dallas et al (2014), Su et al (2017), Nielsen et al (2018)
ELLCPDNT	228 - 235	TRFL_HUMAN	Wada et al (2017)
FKDSAIGF	300 - 307	TRFL_HUMAN	Dallas et al (2014), Su et al (2017), Nielsen et al (2018)
CVDRPVEG	427 - 434	TRFL_HUMAN	Wada et al (2017)
CIGDEQGEN	507 - 515	TRFL_HUMAN	Wada et al (2017)
CLAENAGDVA	534 - 543	TRFL_HUMAN	Wada et al (2017)
NGSDCPDKF	623 - 631	TRFL_HUMAN	Wada et al (2017)
NGSDCPDKFC	623 - 632	TRFL_HUMAN	Wada et al (2017)
TKCELSQ	4 - 10	LALBA_HUMAN	Wada et al (2017)
KDIDGYGGIAL	13 - 23	LALBA_HUMAN	Dallas et al (2014)
GGIALPELI	19 - 27	LALBA_HUMAN	Wada et al (2017)
DTQAIVENNESTE	37 - 49	LALBA_HUMAN	Nielsen et al (2018)
AIVENNESTEY	40 - 50	LALBA_HUMAN	Dallas et al (2014). Nielsen et al (2018)
AIVENNESTEYG	40 - 51	LALBA_HUMAN	Nielsen et al (2018)
IVENNESTEY	41 - 50	LALBA_HUMAN	Dallas et al (2014), Su et al (2017), Nielsen et al (2018)
IVENNESTEYG	41 - 51	LALBA_HUMAN	Su et al (2017), Nielsen et al (2018)
GLFQISNK	51 - 58	LALBA_HUMAN	Nielsen et al (2018)
NICDISCD	71 - 78	LALBA_HUMAN	Wada et al (2017)
NICDISCDK	71 - 79	LALBA_HUMAN	Wada et al (2017)
CDISCDK	73 - 79	LALBA_HUMAN	Wada et al (2017)
DDDITDDIM	82 - 90	LALBA_HUMAN	Wada et al (2017)
ALCTEKLEQ	109 - 117	LALBA_HUMAN	Wada et al (2017)
CTEKLEQ	111 - 117	LALBA_HUMAN	Wada et al (2017)
CTEKLEQWL	111 - 119	LALBA_HUMAN	Wada et al (2017)

TEKLEQW	112 - 118	LALBA_HUMAN	Wada et al (2017)
LQNPSESSEPIPL	12 - 24	CASA1_HUMAN	Nielsen et al (2018)
CAEQFC	84 - 89	CASA1_HUMAN	Wada et al (2017)
SHVQVPFQQL	115 - 124	CASA1_HUMAN	Nielsen et al (2018)
GGFVEGVNK	10 - 18	CEL_HUMAN	Nielsen et al (2018)
ASIDMPAINK	325 - 334	CEL_HUMAN	Nielsen et al (2018)
EDITSHME	171 - 178	OSTP_HUMAN	Nielsen et al (2018)
ELDSASSEVN	289 - 298	OSTP_HUMAN	Nielsen et al (2018)
NNPYVPR	52 - 58	CASK_HUMAN	Wada et al (2017)
LPNSHPPT	79 - 86	CASK_HUMAN	Wada et al (2017)

[1]Without signal peptide
[2]Uniprot entry name

References

Dallas, D.C.; Guerrero, A.; Khaldi, N.; Borghese, R.; Bhandari, A.; Underwood, M.A.; Lebrilla, C.B.; German, J.B.; Barile, D. A Peptidomic Analysis of Human Milk Digestion in the Infant Stomach Reveals Protein-Specific Degradation Patterns. J. Nutr. 2014, 144, 815–820, doi:10.3945/jn.113.185793.

Nielsen, S.D.; Beverly, R.L.; Underwood, M.A.; Dallas, D.C. Release of functional peptides from mother’s milk and fortifier proteins in the premature infant stomach. PLoS One 2018, 13, e0208204, doi:10.1371/journal.pone.0208204.

Beverly, R.L.; Woonnimani, P.; Scottoline, B.P.; Lueangsakulthai, J.; Dallas, D.C. Peptides from the Intestinal Tract of Breast Milk-Fed Infants Have Antimicrobial and Bifidogenic Activity. Int. J. Mol. Sci. 2021, 22, 2377, doi:10.3390/ijms22052377.

Wada, Y.; Phinney, B.S.; Weber, D.; Lönnerdal, B. In vivo digestomics of milk proteins in human milk and infant formula using a suckling rat pup model. *Peptides* **2017**, *88*, 18–31, doi:10.1016/j.peptides.2016.11.012.

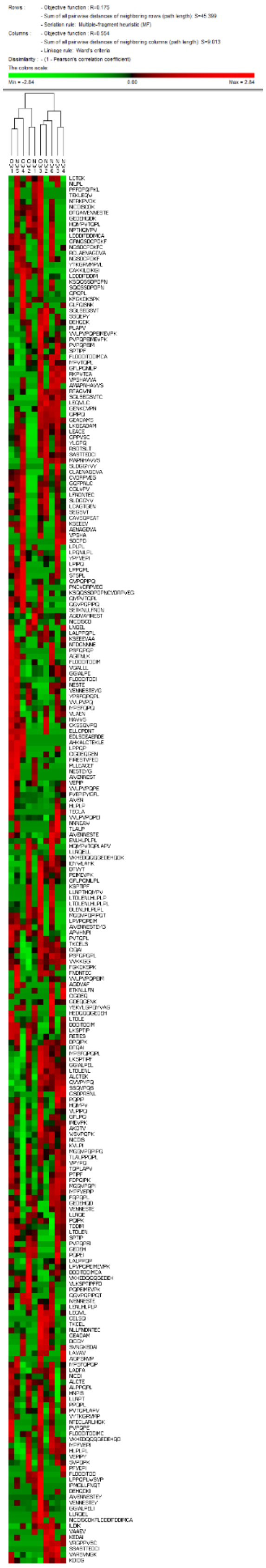

Figure S1. Complete heat map representation of the hierarchical clustering analysis of peptides identified after in vitro digestion of breast milk. Columns correspond to subjects included in the overweight/obese group or normal weight group. Rows correspond to intensity of sequences derived from β-casein, α-lactalbumin and lactoferrin identified in at least 3 subjects.
